# ENIGMA MDD: seven years of global neuroimaging studies of major depression through worldwide data sharing

**DOI:** 10.1038/s41398-020-0842-6

**Published:** 2020-05-29

**Authors:** Lianne Schmaal, Elena Pozzi, Tiffany C. Ho, Laura S. van Velzen, Ilya M. Veer, Nils Opel, Eus J. W. Van Someren, Laura K. M. Han, Lybomir Aftanas, André Aleman, Bernhard T. Baune, Klaus Berger, Tessa F. Blanken, Liliana Capitão, Baptiste Couvy-Duchesne, Kathryn R. Cullen, Udo Dannlowski, Christopher Davey, Tracy Erwin-Grabner, Jennifer Evans, Thomas Frodl, Cynthia H. Y. Fu, Beata Godlewska, Ian H. Gotlib, Roberto Goya-Maldonado, Hans J. Grabe, Nynke A. Groenewold, Dominik Grotegerd, Oliver Gruber, Boris A. Gutman, Geoffrey B. Hall, Ben J. Harrison, Sean N. Hatton, Marco Hermesdorf, Ian B. Hickie, Eva Hilland, Benson Irungu, Rune Jonassen, Sinead Kelly, Tilo Kircher, Bonnie Klimes-Dougan, Axel Krug, Nils Inge Landrø, Jim Lagopoulos, Jeanne Leerssen, Meng Li, David E. J. Linden, Frank P. MacMaster, Andrew M. McIntosh, David M. A. Mehler, Igor Nenadić, Brenda W. J. H. Penninx, Maria J. Portella, Liesbeth Reneman, Miguel E. Rentería, Matthew D. Sacchet, Philipp G. Sämann, Anouk Schrantee, Kang Sim, Jair C. Soares, Dan J. Stein, Leonardo Tozzi, Nic J. A. van Der Wee, Marie-José van Tol, Robert Vermeiren, Yolanda Vives-Gilabert, Henrik Walter, Martin Walter, Heather C. Whalley, Katharina Wittfeld, Sarah Whittle, Margaret J. Wright, Tony T. Yang, Carlos Zarate, Sophia I. Thomopoulos, Neda Jahanshad, Paul M. Thompson, Dick J. Veltman

**Affiliations:** 1 Orygen, The National Centre of Excellence in Youth Mental Health, Parkville, VIC Australia; 2 Centre for Youth Mental Health, The University of Melbourne, Parkville, VIC Australia; 3 Department of Psychology, Stanford University, Stanford, CA USA; 4 Department of Psychiatry & Behavioral Sciences, Stanford University, Stanford, CA USA; 5 Department of Psychiatry & Weill Institute for Neurosciences, University of California, San Francisco, CA USA; 6 Division of Mind and Brain Research, Department of Psychiatry and Psychotherapy CCM, Charité - Universitätsmedizin Berlin, corporate member of Freie Universität Berlin, Humboldt-Universität zu Berlin, and Berlin Institute of Health, Berlin, Germany; 7 Department of Psychiatry, University of Münster, Münster, Germany; 8 Department of Sleep and Cognition, Netherlands Institute for Neuroscience (NIN), an institute of the Royal Netherlands Academy of Arts and Sciences, Amsterdam, Netherlands; 9 Department of Integrative Neurophysiology, Center for Neurogenomics and Cognitive Research (CNCR), Amsterdam Neuroscience, VU University Amsterdam, Amsterdam, The Netherlands; 10 Department of Psychiatry, Amsterdam UMC, Vrije Universiteit Amsterdam, Amsterdam Neuroscience, Amsterdam Public Health Research Institute, Amsterdam, The Netherlands; 11 FSSBI Scientific Research Institute of Physiology & Basic Medicine, Laboratory of Affective, Cognitive & Translational Neuroscience, Novosibirsk, Russia; 12 Department of Neuroscience, Novosibirsk State University, Novosibirsk, Russia; 13 Department of Biomedical Sciences of Cells and Systems, University Medical Center Groningen, University of Groningen, Groningen, The Netherlands; 14 Department of Psychiatry, University of Melbourne, Melbourne, VIC Australia; 15 The Florey Institute of Neuroscience and Mental Health, University of Melbourne, Melbourne, VIC Australia; 16 Institute of Epidemiology and Social Medicine, University of Münster, Münster, Germany; 17 Department of Psychiatry, Oxford University, Oxford, UK; 18 Oxford Health NHS Foundation Trust, Oxford, UK; 19 Institute for Molecular Bioscience, the University of Queensland, Brisbane, QLD Australia; 20 Department of Psychology, University of Minnesota, Minneapolis, MN USA; 21 Laboratory of Systems Neuroscience and Imaging in Psychiatry (SNIP-Lab), University Medical Center Göttingen, Göttingen, Germany; 22 Experimental Therapeutics Branch, NIMH, NIH, Bethesda, MD USA; 23 Department of Psychiatry and Psychotherapy, Jena University Hospital, Jena, Germany; 24 School of Psychology, University of East London, London, UK; 25 Centre for Affective Disorders, Institute of Psychiatry, Psychology and Neuroscience, King’s College London, London, UK; 26 Department of Psychiatry and Psychotherapy, University Medicine Greifswald, Greifswald, Germany; 27 German Center for Neurodegenerative Diseases (DZNE), Site Rostock/Greifswald, Germany; 28 Department of Psychiatry & Mental Health, University of Cape Town, Cape Town, South Africa; 29 Section for Experimental Psychopathology and Neuroimaging, Department of General Psychiatry, Heidelberg University Hospital, Heidelberg, Germany; 30 Illinois Institute of Technology, Chicago, IL USA; 31 Department of Psychology, Neuroscience & Behaviour, McMaster University, Hamilton, ON Canada; 32 Melbourne Neuropsychiatry Centre, Department of Psychiatry, The University of Melbourne & Melbourne Health, Melbourne, VIC Australia; 33 Brain and Mind Centre, University of Sydney, Camperdown, NSW Australia; 34 Clinical Neuroscience Research Group, Department of Psychology, University of Oslo, Oslo, Norway; 35 Department of Psychiatry, Diakonhjemmet Hospital, Oslo, Norway; 36 Norwegian Centre for Mental Disorders Research (NORMENT), Institute of Clinical Medicine, University of Oslo, Oslo, Norway; 37 Department of Psychiatry & Behavioral Sciences, The University of Texas Health Science Center at Houston, Houston, TX USA; 38 Faculty of Health Sciences, Oslo Metropolitan University, Oslo, Norway; 39 Beth Israel Deaconess Medical Centre, Harvard Medical School, Boston, MA USA; 40 Department of Psychiatry and Psychotherapy, University of Marburg, Marburg, Germany; 41 Sunshine Coast Mind and Neuroscience Thompson Institute, University of the Sunshine Coast, Birtinya, QLD Australia; 42 Division of Psychological Medicine and Clinical Neurosciences, Cardiff University, Cardiff, UK; 43 MRC Center for Neuropsychiatric Genetics and Genomics, Cardiff University, Cardiff, UK; 44 Cardiff University Brain Research Imaging Center, Cardiff University, Cardiff, UK; 45 Psychiatry and Pediatrics, University of Calgary, Addictions and Mental Health Strategic Clinical Network, Calgary, AB Canada; 46 Centre for Clinical Brain Science, University of Edinburgh, Edinburgh, UK; 47 Marburg University Hospital UKGM, Marburg, Germany; 48 Institut d’Investigació Biomèdica-Sant Pau, Barcelona, Spain; 49 CIBERSAM, Madrid, Spain; 50 Universitat Autònoma de Barcelona, Barcelona, Spain; 51 Department of Radiology and Nuclear Medicine, location AMC, Amsterdam UMC, Amsterdam, The Netherlands; 52 Department of Genetics & Computational Biology, QIMR Berghofer Medical Research Institute, Brisbane, QLD Australia; 53 Center for Depression, Anxiety, and Stress Research, McLean Hospital, Harvard Medical School, Belmont, MA USA; 54 Max Planck Institute of Psychiatry, Munich, Germany; 55 West Region/Institute of Mental Health, Singapore, Singapore; 56 Yong Loo Lin School of Medicine/National University of Singapore, Singapore, Singapore; 57 SA MRC Research Unit on Risk & Resilience in Mental Disorders, Department of Psychiatry & Neuroscience Institute, University of Cape Town, Cape Town, South Africa; 58 Department of Psychiatry, Leiden University Medical Center, Leiden, The Netherlands; 59 Leiden Institute for Brain and Cognition, Leiden University Medical Center, Leiden, The Netherlands; 60 Curium-LUMC, Leiden University Medical Center, Leiden, The Netherlands; 61 Instituto ITACA, Universitat Politècnica de València, Valencia, Spain; 62 Department of Psychiatry and Psychotherapy, Jena, Germany; 63 Clinical Affective Neuroimaging Laboratory, Leibniz Institute for Neurobiology, Magdeburg, Germany; 64 Queensland Brain Institute, The University of Queensland, Brisbane, QLD Australia; 65 Centre for Advanced Imaging, The University of Queensland, Brisbane, QLD Australia; 66 Section on the Neurobiology and Treatment of Mood Disorders, National Institute of Mental Health, Bethesda, MD USA; 67 Imaging Genetics Center, Mark and Mary Stevens Neuroimaging and Informatics Institute, Keck School of Medicine, University of Southern California, Marina del Rey, CA USA

**Keywords:** Depression, Neuroscience

## Abstract

A key objective in the field of translational psychiatry over the past few decades has been to identify the brain correlates of major depressive disorder (MDD). Identifying measurable indicators of brain processes associated with MDD could facilitate the detection of individuals at risk, and the development of novel treatments, the monitoring of treatment effects, and predicting who might benefit most from treatments that target specific brain mechanisms. However, despite intensive neuroimaging research towards this effort, underpowered studies and a lack of reproducible findings have hindered progress. Here, we discuss the work of the ENIGMA Major Depressive Disorder (MDD) Consortium, which was established to address issues of poor replication, unreliable results, and overestimation of effect sizes in previous studies. The ENIGMA MDD Consortium currently includes data from 45 MDD study cohorts from 14 countries across six continents. The primary aim of ENIGMA MDD is to identify structural and functional brain alterations associated with MDD that can be reliably detected and replicated across cohorts worldwide. A secondary goal is to investigate how demographic, genetic, clinical, psychological, and environmental factors affect these associations. In this review, we summarize findings of the ENIGMA MDD disease working group to date and discuss future directions. We also highlight the challenges and benefits of large-scale data sharing for mental health research.

## Introduction

Major depressive disorder (MDD) is the largest contributor to the disease burden caused by poor mental health worldwide owing to its high prevalence, high recurrence rates, chronicity, and comorbidity with physical illness^1^. Thus, effective and early treatment is crucial. Unfortunately, current strategies for treating MDD, which do not take neurobiological markers into consideration, have not been particularly effective^2–6^.

Over the past several decades, technical advances in neuroimaging have provided the impetus for identifying measurable indicators of brain processes associated with MDD in order to detect individuals at risk for the disorder, to facilitate the development of novel interventions, and to evaluate treatment effects. Although several neuroimaging markers have been found that differentiate patients with MDD from healthy controls (e.g., Mulders et al.^7^; Kempton et al.^8^), progress has still been limited in part by underpowered studies and a lack of reproducible findings (e.g., Kapur et al.^9^). Many research studies in the field have been restricted by small samples, resulting in a lower probability of finding “true” effects (low-powered studies tend to produce more false negatives than do high-powered studies) and an inflated estimate of the effect size when a true effect is discovered^10–12^. This is particularly problematic when the true effect size is modest, which is often the case for differences in brain imaging measures between patients and controls. Underpowered studies also make it difficult to reproduce significant findings, leading to inconsistent and poorly replicated neuroimaging findings in depression^13,14^.

Larger samples and meta-analytic approaches represent good strategies to overcome issues associated with small sample sizes. Large-scale data collection initiatives with harmonized assessments (including neuroimaging), such as the population-based UK Biobank study (*N* = 500,000)^15^, are yielding key insights into brain mechanisms involved in MDD (e.g., Howard et al.^16^; Shen et al.^17^; Harris et al.^18^). However, recruiting large samples is not always feasible because of limited access to patient populations at any one site or limited availability of scanning facilities and the financial costs of scanning hundreds or even thousands of participants. Moreover, large-scale population-based samples also typically focus on individuals from a single geographic region or country within a restricted age range, thereby limiting the generalizability of findings across countries, cultures, and developmental stages. Issues with retrospective meta-analyses include the potential over-representation of positive findings in the published literature (publication bias) and a lack of harmonization of data processing and statistical analysis methods across the different studies included in the meta-analysis.

Worldwide pooling of existing neuroimaging data offer a highly effective alternative to larger non-generalizable studies and retrospective meta-analyses, as it (1) makes optimal use of valuable and costly existing data sets from individual studies; (2) collates large data sets at a relatively low cost; (3) allows coordinated analysis using standardized protocols for data processing and analysis; and (4) combines expertize of hundreds of professionals in the fields of neuroimaging, psychiatry, statistics, and mathematics. Here, we discuss the work of the worldwide Enhancing NeuroImaging Genetics through Meta-Analysis (ENIGMA) Major Depressive Disorder (MDD) consortium.

## The ENIGMA MDD consortium

The MDD Working Group was founded in 2012 as part of the ENIGMA consortium. ENIGMA was initiated in 2009 to boost statistical power in genome-wide association studies (GWAS) that aimed to identify common genetic variants that affect brain structure^19–21^. Because most major mental illnesses have a high-dimensional genetic architecture with polygenic influences, epistasis and gene by environment interactions, by focusing on intermediate phenotypes—or endophenotypes—at the level of MRI-derived brain measures, it was thought that researchers would be better able to identify the neurobiological underpinnings of psychiatric disorders^22–24^. Therefore, the ENIGMA consortium was launched to combine existing genomic and neuroimaging data around the world to conduct well-powered GWAS analyses. ENIGMA has since published the largest genetic studies of the brain, in partnership with other consortia^25–30^, mapping genome-wide effects of over a million genetic loci in over 30,000–50,000 brain MRI scans (for a recent review of the ENIGMA imaging genetics findings, see Thompson et al.^21^).

Building on ENIGMA’s initial successes in imaging genetics, disease working groups were formed to study patterns of brain abnormalities in major psychiatric, neurodevelopmental, neurological, and neurogenetic disorders. ENIGMA MDD was established with the initial aim to (1) identify structural and functional brain alterations associated with MDD that can be reliably detected and replicated across many different samples worldwide; and (2) identify demographic, genetic, clinical, psychological, and environmental factors that affect these associations.

**Fig. 1:**
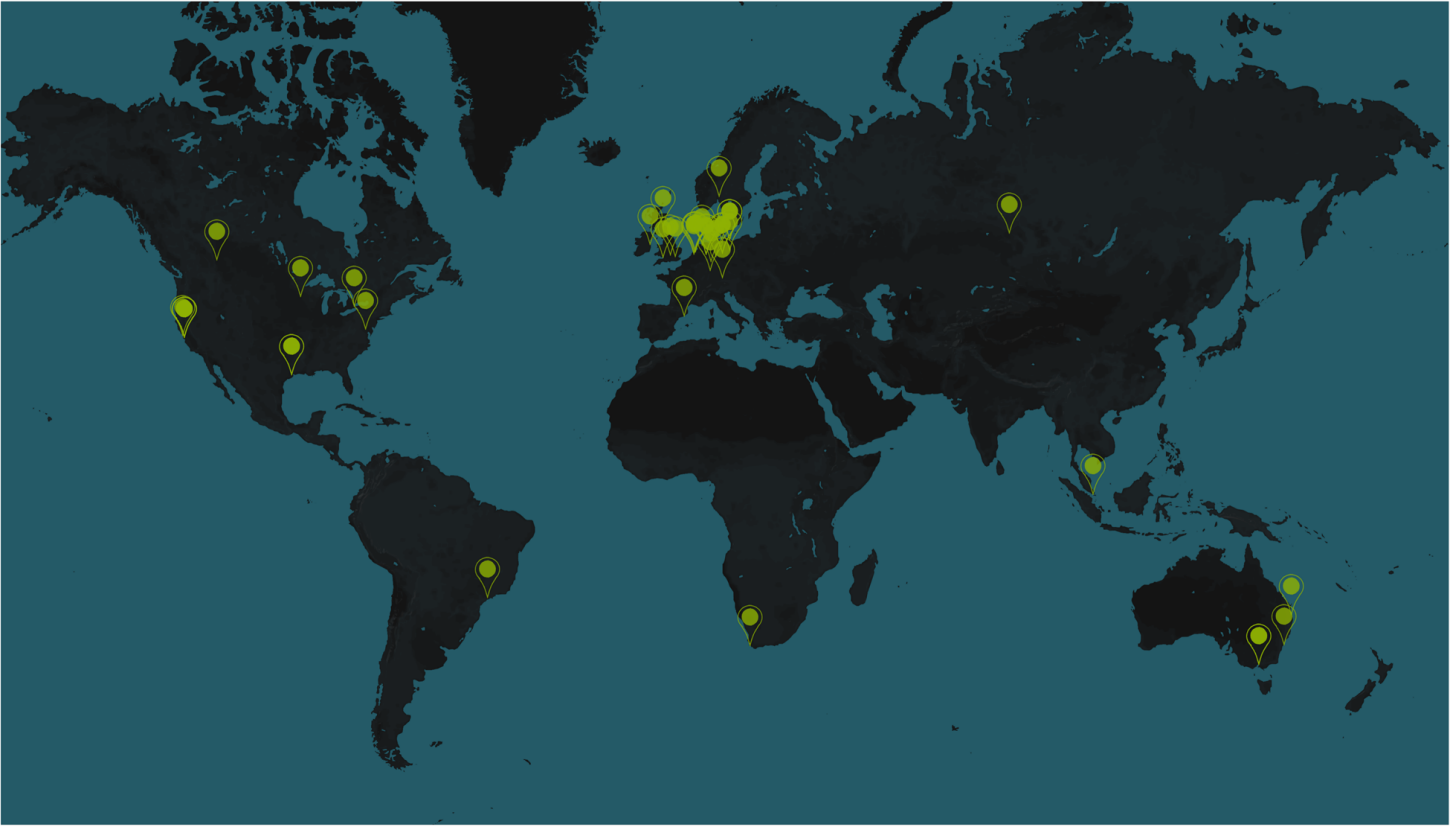
World map of cohorts participating in ENIGMA MDD. Locations of cohorts included in the ENIGMA MDD consortium in September 2019.

Since it was established, ENIGMA MDD has grown to 35 participating research institutions (including 45 study cohorts) from 14 different countries across six continents in September 2019 (Fig. 1). For an up to date overview of all participating research institutions, see: http://enigma.ini.usc.edu/wp-content/uploads/2019/10/List_members_oct2019-2.pdf. To date, participating researchers have shared demographic, clinical and neuroimaging data from 9788 healthy individuals and 4372 individuals with MDD.

**Fig. 2:**
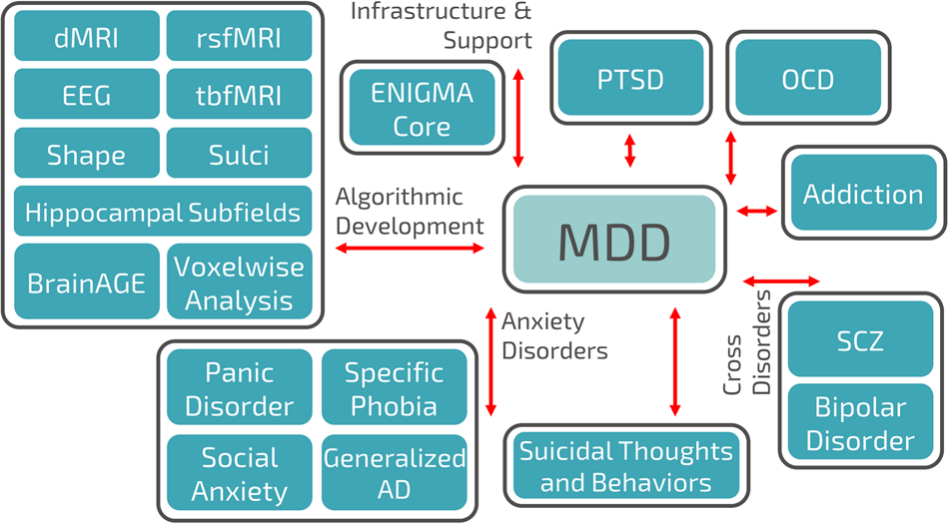
Connections between ENIGMA MDD and other ENIGMA working groups. Note: not all ENIGMA working groups are displayed in this figure. In September 2019, ENIGMA includes 50 working groups, of which 26 working groups focus on mental and neurological disorders. dMRI diffusion magnetic resonance imaging (MRI), rsfMRI resting state functional MRI, EEG electroencephalogram, tbfMRI task-based functional MRI, MDD major depressive disorder, PTSD post-traumatic stress disorder, AD anxiety disorder.

ENIGMA MDD and other disease working groups are supported by the ENIGMA Methods working groups (Fig. 2), which are dedicated to developing standardized processing, quality assurance, and statistical analysis protocols, to reducing statistical heterogeneity and researcher degrees of freedom, and to ensuring or evaluating reproducibility. Brain measures derived from the ENIGMA protocols have shown good reliability^31–34^. Because ENIGMA is dedicated to “open science”, all ENIGMA protocols are publicly available on the ENIGMA website.

Here, we discuss findings of structural brain alterations associated with MDD and depression-related phenotypes that were obtained through this worldwide data-sharing initiative. We also discuss clinical implications, future directions for the ENIGMA MDD consortium and identify challenges of large-scale data sharing.

## ENIGMA MDD findings to date

The initial studies conducted with data available through the ENIGMA MDD consortium focused on identifying associations between MDD and structural brain measures that could reliably be detected across many samples worldwide. Because inconsistent findings across previous retrospective meta-analyses may be owing in part to differences in data processing and statistical analyses among the primary studies, we conducted individual participant data (IPD)-based (or prospective) meta-analyses to identify differences in subcortical volume, subcortical shape, cortical thickness, cortical surface area, and white matter integrity between patients with MDD and healthy controls, and to examine the effects of demographic and clinical characteristics^35–38^. Harmonized imaging processing (e.g., Freesurfer^39^), quality assurance (http://enigma.ini.usc.edu/protocols/imaging-protocols/) and statistical analysis protocols were run locally on data from participating cohorts. In addition, the scope of retrospective meta-analyses in terms of examined brain regions is limited to those reported in the original studies, as many published studies adopted a hypothesis-driven approach and focused on specific regions of interest (ROIs). The large sample size available in ENIGMA MDD ensures the statistical power needed to investigate whole-brain structural brain alterations. For the IPD-based meta-analyses, summary statistics of each site were shared to be included in a random effects meta-analysis to examine differences in structural brain measures between MDD patients and controls.

A few ENIGMA MDD studies have started to adopt a mega-analytic approach, where individual-level measures derived from the harmonized imaging processing protocols are pooled across sites and regression analyses are conducted on this pooled dataset while correcting for confounding site effects (e.g., linear mixed models with a random intercept for site). Key advantages of a meta-analytic versus mega-analytic approach include: (1) allowing the analysis of individual studies to account for local population substructure; (2) allowing analysis of study-specific covariates that may be better dealt with within each study; and (3) allowing analyses to be conducted within each participating site and results to be shared through a central site without requiring individual-level data to be shared^40^. However, the advantages of a mega-analytic versus meta-analytic approach include (1) greater flexibility in the control of confounders at the level of individual patients and studies; and (2) not having to assume within-study normality and known within-study variances^40^, as these assumptions can be especially problematic with smaller samples^41^. Moreover, pooling all data in a single statistical model may boost statistical power to detect certain effects^42^, such as higher-level interactions, when the phenotype of interest is rare (e.g., suicide attempt, number of medication naive patients) or when the range of continuous variables is limited (e.g., age, symptom severity, childhood trauma scores) in individual studies. All ENIGMA MDD studies published to date are summarized in Table 1.

**Table 1:** Overview of ENIGMA MDD studies published to date.

Study	Modality	Meta- or mega-analysis	Sample size	No. of cohorts	Main findings
Schmaal et al. 2016 in Molecular Psychiatry	sMRI (FreeSurfer subcortical volumes)	Meta	HC: 7199 MDD: 1728	15	Mean hippocampal volume was significantly lower in MDD compared with HC. This effect was driven primarily by recurrence of MDD (i.e., >1 episode). MDD with an early age of onset (⩽21 years) showed significantly lower mean hippocampal volumes than HC.
Schmaal et al. 2017 in Molecular Psychiatry	sMRI (FreeSurfer cortical thickness and surface area)	Meta	Adults: HC: 7658 MDD: 1902 Adolescents: HC: 294 MDD: 213	20	Compared with adult HC, adults with MDD showed lower cortical thickness in the bilateral medial OFC, cingulate cortex, insula and temporal lobes, but no surface area alterations. Subgroup analysis revealed lower cortical thickness in adults with MDD with adult onset, but not adolescent onset, age of onset, relative to HC. Compared with adolescent HC, adolescents with MDD had lower total surface area (but no differences in cortical thickness), with most pronounced effects in medial OFC, superior frontal gyrus, and primary and higher order visual, somatosensory and motor areas. These effects were driven by adolescents with recurrent depression.
Renteria et al. 2017 in Translational Psychiatry	sMRI (FreeSurfer subcortical volumes)	Meta	HC: 1996 MDD: 1101	20	No significant differences were found between MDD with suicidal ideation and HC. MDD with suicidal behavior (reported suicidal attempts or plans) showed a trend toward significant smaller ICV, compared with HC. No significant differences were found between MDD with and without suicidal ideation and/or behavior.
Frodl et al. 2017 in Journal of Psychiatry Research	sMRI (FreeSurfer subcortical volumes)	Mega	HC: 2078 MDD: 958	9	Severity of childhood maltreatment (CM) was associated with lower caudate volumes in females, but no significant effects were found in males. The effect was associated with all subcategories of CM, but most pronounced for childhood emotional and physical neglect. The effect was independent of MDD diagnosis.
Tozzi et al. 2019 in Psychological Medicine	sMRI (FreeSurfer cortical thickness and surface area)	Mega	HC: 2588 MDD: 1284	12	Regardless of MDD, overall severity of childhood maltreatment (CM) was associated with lower thickness in the supramarginal gyrus, banks of the superior temporal sulcus and lower surface area in the middle temporal lobe. Compared with no CM, the combination of childhood abuse and neglect showed lower cortical thickness in the same areas, in addition to the inferior parietal lobe, middle temporal lobe, and precuneus, whereas no effects were found for abuse or neglect alone. Males—but not females—with MDD and a history of CM showed greater surface area in the rostral ACC compared with the no CM group. The negative association between CM severity and thickness of various prefrontal, cingulate, and temporal regions was more pronounced with increasing age. No significant interaction effect between MDD diagnosis and CM.
de Kovel et al. 2019 in American Journal of Psychiatry	sMRI (FreeSurfer subcortical volumes, cortical thickness and surface area)	Mega	Cortical regions: HC: 3504 MDD: 2256 Subcortical regions: HC: 4230 MDD: 2540	Cortical regions: 31 Subcortical regions: 32	No differences in the laterality of cortical regions thickness and surface area or subcortical volumes were found between MDD and HC.
Ho et al. 2020 in Human Brain Mapping	sMRI (FreeSurfer subcortical shapes)	Meta	HC: 2953 MDD: 1781	10	Compared with HC, MDD had lower thickness and surface area in the subiculum and CA2/3 areas of the hippocampus and basolateral amygdala. These effects were primarily driven by MDD with an adolescent age of onset (⩽21 years). Recurrence of MDD was associated with lower surface area and thickness in the basolateral amygdala and in the CA1 region of the hippocampus.
Han et al. 2020 in Molecular Psychiatry	sMRI (FreeSurfer subcortical volumes, cortical thickness and surface area)	Mega	HC: 4314 MDD: 2675	19	Compared with HC, MDD showed higher brain-PAD (brain-predicted age difference of 1.08 years). Strongest effects were found in MDD using antidepressants at time of scanning, patients in an active episode and patients in remission compared with HC, but there were no significant differences between the MDD subgroups. Brain-PAD was positive in all MDD subgroups, indicating that individuals with MDD were estimated to be older than expected based on the brain age model.
Van Velzen et al. 2019 in Molecular Psychiatry	DTI (FA, RD, MD and AD for atlas-defined white matter tracts of interest)	Meta	Adults: HC: 1265 MDD: 921 Adolescents: HC: 290 MDD: 372	20	Adults with MDD showed lower FA in 16 of the 25 WM tracts examined, relative to HC. These effects appeared to be global, with the corona radiate and the corpus callosum contributing most. The effects were mainly driven by recurrent MDD, MDD with adult age of onset (>21 years) and antidepressant-free patients at the time of scanning. Higher RD in adults with MDD was also observed across different ROIs. No differences were found between healthy adolescents and adolescents with MDD.

*DTI* Diffusion tensor imaging, *FA* fractional anisotropy, *HC* healthy controls, *ICV* intracranial volume, *MDD* major depressive disorder, *CM* childhood maltreatment, *OFC* orbitofrontal cortex, *ACC* anterior cingulate cortex, *RD* radial diffusivity, *MD* mean diffusivity, *AD* axial diffusivity, *ROIs* regions of Interest, *Brain-PAD* brain-predicted age differencefractional anisotropy, *sMRI* structuralmagnetic resonance imaging, *WM* white matter.

### Structural brain alterations in MDD

#### Subcortical brain regions

**Fig. 3:**
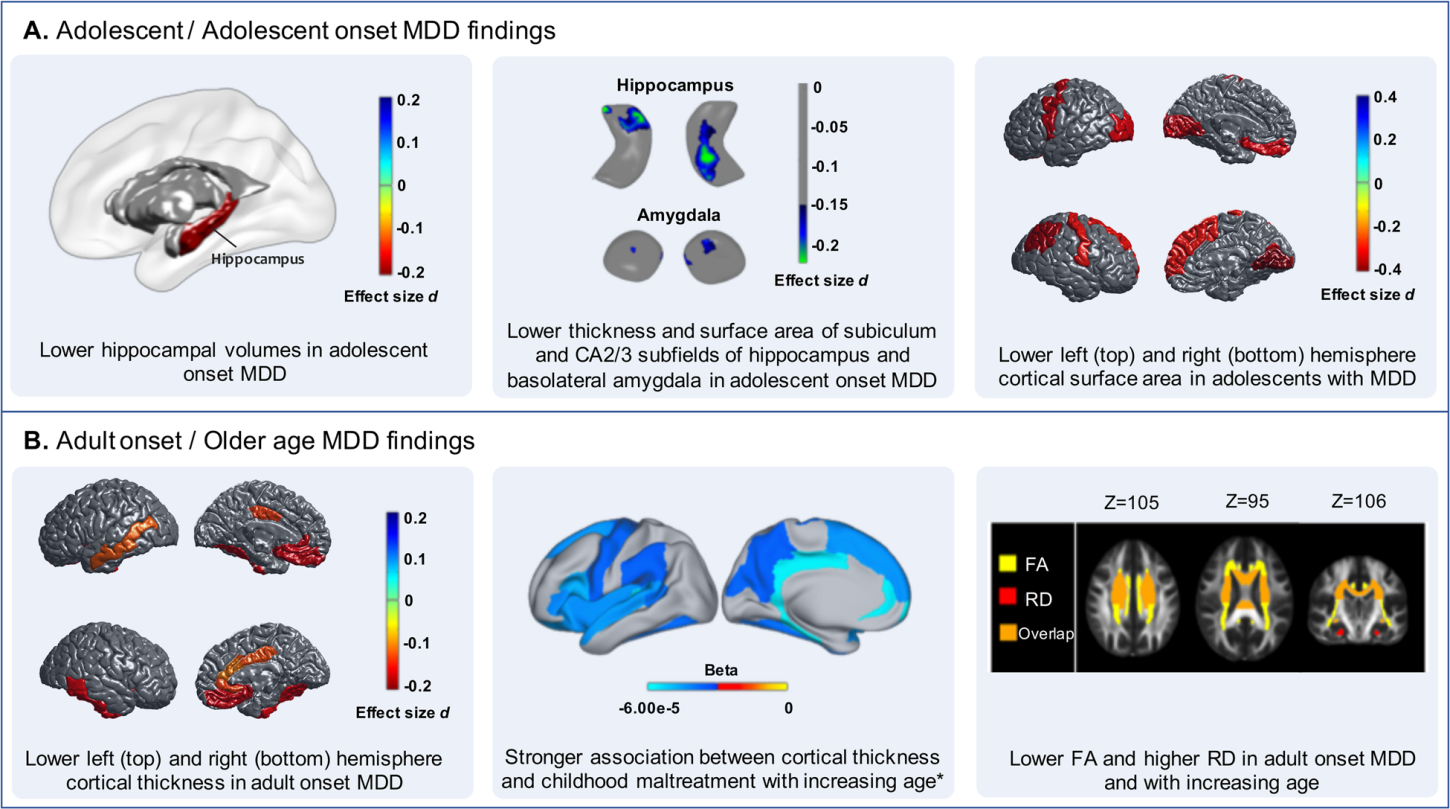
Converging findings across ENIGMA MDD studies. Specific characteristics of brain structure are differentially affected by MDD (or vice versa) at different stages of life. **a** Alterations in hippocampal and amygdala volumes and shapes are observed in adolescent-onset MDD and lower cortical surface area in adolescents with MDD. **b** Cortical thickness alterations and white matter abnormalities are specifically associated with adult-onset MDD and older age in individuals with MDD and/or childhood maltreatment. *This association was independent of MDD diagnosis. MDD major depressive disorder, FA fractional anisotropy, RD radial diffusivity.

The first ENIGMA MDD project focused on differences in subcortical volume between MDD patients (*N* = 1728) and healthy controls (*N* = 7199)^35^. Consistent with prior studies and retrospective meta-analyses^8,43–45^, in this IPD-based meta-analysis we found significantly lower hippocampal volumes in individuals with MDD compared with healthy controls. This effect was also consistently observed across individual cohorts, although the overall effect size was modest (Cohen’s *d* = −0.14). The hippocampal volume deficit was greater in MDD patients with recurrent episodes (*N* = 1119, Cohen’s *d* = −0.17), compared with healthy controls, whereas no hippocampal volume alterations were observed in first-episode patients (*N* = 583). Our findings may suggest that depression-related reductions in hippocampal volume are a result of longer illness duration or greater number of episodes, instead of a premorbid vulnerability factor. This is consistent with prior longitudinal studies showing greater hippocampal atrophy in individuals with persistent, recurring, or worsening of depressive symptoms over time^46–49^. Nonetheless, it is unclear whether hippocampal atrophy associated with prolonged illness duration or recurrence represents a state marker instead of a permanent scar. Fortunately, hippocampal alterations may normalize as hippocampal enlargement has been observed following remission or treatment of MDD^48,50,51^. We also found smaller hippocampal volumes in patients with an adolescent onset of MDD (⩽21 years; *N* = 541; Cohen’s *d* = −0.20), compared with controls, whereas no differences were observed in those with an adult onset of MDD (*N* = 997) (Fig. 3a). This is in line with previous studies showing smaller hippocampal volumes in adolescents and even children with depression^52–55^, whereas other studies found lower hippocampal volumes only in adults with an age of onset >30^56^ or no differences in hippocampal volume between adults with an adolescent versus adult age of onset of MDD^57^. Because only about half (57%) of the adolescent-onset patients had a recurrent episode of MDD, adolescent disease onset may, in part, have an independent association with hippocampal volumes. Smaller hippocampal volumes may precede disease onset, especially in this early-onset group, perhaps as a result of factors commonly associated with early-onset MDD including childhood adversity^58,59^ and genetic influences^60^. Longitudinal studies designed to track hippocampal volume changes prior to disease onset and over the disease course are required to elucidate whether hippocampal abnormalities result from a prolonged duration of chronic stress associated with depressive episodes, represent a vulnerability factor for MDD, or both.

Interestingly, we did not detect significant differences for any of the other subcortical volumes, including the amygdala, nucleus accumbens, caudate, putamen, thalamus, and pallidum, or the lateral ventricles and intracranial volume (ICV). Previous reports have varied regarding volume abnormalities in subcortical regions other than the hippocampus, such as the amygdala^8,61,62^. Nonetheless, associations with MDD may still be present for functionally distinct subregions within these broader subcortical regions. Patterns of depression-related alterations in subregions of subcortical surfaces have been difficult to identify as there are few identifiable surface landmarks, in contrast with the landmarks consistently found in cortical surfaces (e.g., deep sulcal patterns). In this context, subcortical shape analysis may be a more sensitive method to identify more localized effects in subdivisions in subcortical regions that were not captured by the volumetric analysis of subcortical regions in the first paper from the ENIGMA MDD consortium^35^. To address this, we conducted an additional multi-site meta-analytic investigation to test whether MDD patients, and specific subgroups of MDD based on important clinical characteristics, differ from healthy controls in subcortical shape. Specifically, we applied meta-analytic models on effect sizes generated from 1781 patients with MDD and 2953 healthy controls across 10 study cohorts. Consistent with the findings from our first meta-analysis, we found that relative to healthy controls (*N* = 2879), patients with an adolescent onset of MDD (*N* = 476) had lower thickness (Cohen’s *d* = −0.17) and smaller surface area (Cohen’s *d* = −0.18) in the hippocampus, with most pronounced effects in the subiculum and *cornu ammonis* (CA) subfields two and three of the hippocampus^37^ (Fig. 3a). Extending our prior findings, we also observed lower thickness (Cohen’s *d* = −0.16) and smaller surface area (Cohen’s *d* = −0.17) in the amygdala in adolescent-onset patients, specifically within the basolateral subdivision of the amygdala^37^ (Fig. 3a). These subregions are rich in glucocorticoid receptors, emphasizing that disturbed glucocorticoid signaling during stress response promotes the development of MDD^63^. Importantly, shape analyses of subcortical structures clarify results in the extant literature of smaller hippocampal volumes and ambiguous effects in the amygdala in patients with MDD; delineating nuanced effects in depression-related subregions of subcortical structures may help to identify more precise intervention targets or more sensitive biomarkers of treatment response. Noteworthy, additional ENIGMA MDD analyses regarding associations between MDD and FreeSurfer-derived hippocampal subfields are currently underway.

#### Cortical thickness and surface area

Following these studies of subcortical morphology, we examined cortical thickness and surface area in relation to MDD and clinical characteristics in a meta-analysis of data from 20 participating ENIGMA MDD cohorts^36^. Because more research groups joined ENIGMA MDD after the publication of our subcortical volume meta-analysis study, we were able to conduct separate analyses in young people (⩽21 years) and adults (>21 years). Most published studies to date have focused on regional cortical volume, which is a function of cortical thickness and surface area. Advances in neuroimaging data processing have made it possible to separate cortical surface area and cortical thickness, which is important to do in the context of understanding brain correlates of MDD, given that these neural characteristics are genetically and phenotypically distinct^27,64,65^. In adults, we observed subtle cortical thickness alterations in 13 of 68 cortical regions in patients with MDD (*N* = 1902) compared with healthy controls (*N* = 7658) (Cohen’s *d*’s between −0.10 and −0.14), including lower thickness of the bilateral medial orbitofrontal cortex (OFC), fusiform gyrus, insula, rostral anterior (ACC) and posterior cingulate cortex (PCC) and unilaterally in the left middle temporal gyrus, right inferior temporal gyrus and right caudal ACC (Fig. 3b). Our findings in adults with MDD were consistent with prior meta-analyses showing depression-related structural alterations in the medial PFC and ACC^8,66–69^; however, they extended previous findings by demonstrating structural abnormalities in the temporal regions (middle and inferior temporal gyri and fusiform gyrus), posterior cingulate cortex and insula. These cortico-limbic thickness alterations may contribute to the broad spectrum of emotional, cognitive, and behavioral disturbances in MDD.

The largest effect size was observed in the medial OFC, which—in contrast to lower hippocampal volume—was already detectable in first-episode patients. In contrast to the hippocampal volume finding that were driven by adult patients with an adolescent onset of their first depressive episode, the lower cortical thickness findings were driven mostly by adult patients with an adult onset of MDD (*N* = 1214; Cohen’s *d* −0.11 to −0.18) relative to controls. No cortical surface area differences were found among the adult groups. We speculated that the more pronounced effects in adult patients with an adult age of onset may be driven in part by their older age compared with adult patients with an adolescent age of onset of their first depressive episode, which was confirmed by a post hoc moderator analysis with mean age of patients in each sample. This suggests that mental illness has a greater impact on cortical thickness in the context of aging. Indeed, cortical thickness has been shown to be a more sensitive indicator of aging than is surface area or volume^70–72^. Because our meta-analytic approach did not allow us to pool all data across samples, we were not able to investigate age-by-diagnosis effects across the entire age range (individual samples had restricted age ranges). Therefore, future mega-analyses could further elucidate these dynamic relations with development and aging.

Surprisingly, in contrast to adults with MDD, adolescents with MDD showed no cortical thickness alterations, but rather, alterations in global cortical surface area (Fig. 3a); the effect sizes of these results were larger (Cohen’s *d* −0.31 to −0.41 in local regions) than the cortical thickness alterations observed in adults. Cortical surface area has been understudied in the context of MDD. Nonetheless, a recent longitudinal study showed that lower surface area was specifically observed in young people experiencing depressive symptoms in early adolescence but not in those developing depressive symptoms later in adolescence, and that lower surface area was already observable in young people with subclinical depressive symptoms, not all of whom will develop a full-threshold MDD diagnosis^36^. Thus, cortical surface area reductions may represent an early developing subtype of depressive disorder, shaped by genetic factors or early life adversity (prenatal^73,74^ or perinatal or during childhood^75–77^), and potentially precede the onset of MDD. This hypothesis is consistent with the observation that, compared with cortical thickness, cortical surface area has a higher genetic heritability^27,64,78^, has a genetic correlation with MDD and depressive symptoms (this genetic association is absent for cortical thickness^27^), is determined earlier in development, and is less strongly affected by later environmental influences^71,79^.

Importantly and paradoxically, no differences in surface area were observed in adult patients with adolescent-onset MDD. This might be explained by (1) normalization of cortical surface area when transitioning into adulthood; (2) cortical surface area alterations being only present in a specific subgroup (subtype) of adult patients with adolescent-onset MDD, which we were unable to detect; or (3) those with cortical surface area alterations in early adolescence may be at higher risk for transitioning from MDD to other mental disorders over time. This latter possibility is consistent with reports of lower cortical surface area in adolescents and adults with psychosis or schizophrenia^80,81^, and in individuals at high risk for and/or transitioning to psychosis^82,83^. Longitudinal studies are required to test the hypothesis that cortical surface area alteration is a pre-existing risk factor for the development of MDD, and to investigate the subsequent clinical course of these depressed young people with global surface area reductions.

#### Brain asymmetry

Altered brain asymmetry may have a role in MDD, especially at a functional level^84^, but only a few studies have investigated structural asymmetry^85,86^. In our studies, the majority of cortical thickness or surface area alterations were observed across both hemispheres, with a few regions showing only unilateral differences^36^. However, we did not explicitly test whether the effect sizes of our findings differed significantly by hemisphere. Therefore, in a separate study, we investigated structural asymmetries by investigating asymmetry indices ((left − right)/(left + right)) for local and global cortical and subcortical brain regions in individuals with MDD (*N* = 2256) compared with healthy controls (*N* = 3504)^87^. The results showed no significant differences in brain structural asymmetry between individuals with MDD and controls for any of the structural brain measures, nor any associations with clinical characteristics. These findings suggest that altered brain structural asymmetry is of little relevance to the pathophysiology of MDD, although functional asymmetries may still play a role.

#### Brain aging in MDD

MDD is associated with an increased risk of aging-related medical illnesses such as cardiovascular disease and cancer^88,89^. Although aging is associated with loss of gray matter, depression may accelerate age-related brain atrophy^90^. Therefore, we examined deviations from normative brain aging in adults with MDD and associated clinical heterogeneity by pooling data from >6900 healthy controls and individuals with MDD from 19 different scanners participating in the ENIGMA MDD consortium^91^. Normative brain aging was estimated by predicting chronological age (18–75 years) from 7 subcortical volumes, 34 cortical thickness and 34 surface area, lateral ventricles and ICV measures using Ridge Regression, separately in 952 male and 1236 female controls. We showed that our brain age prediction model generalized to unseen hold-out samples (927 male controls and 986 males with MDD, and 1199 female controls and 1689 females with MDD; correlations *r* between predicted and actual age ranged from 0.77–0.85, mean absolute errors (MAE) ranged from 6.32 to 7.18 years), as well as to completely independent samples from different scanning sites (*N* = 1330 from 23 different scanners; *r* = 0.71 and MAE = 7.49 for male controls, *r* = 0.72 and MAE = 7.26 for female controls)^91^.

Brain-predicted age difference (brain-PAD) was computed from the difference between predicted “brain age” and chronological age^92^. We found that, at the group level, MDD patients had a + 1.08 years (Cohen’s *d* = 0.14, *p* < 0.0001) greater discrepancy between their predicted and actual age compared with control participants. In other words, individuals with MDD were estimated to be ~ 1 year older than expected based on the brain age model. The brain age model relied mostly on cortical thickness measures (compared with subcortical volumes, cortical surface area and ICV) in order to make good age predictions. Brain-PAD differences were observed in all subgroups of patients compared with controls, with no significant differences in brain-PAD between the patient groups.

As many of the MDD patients did not show advanced brain aging compared with controls, the clinical significance of the observed higher brain-PAD in MDD patients may be limited. However, there may still be a subgroup of MDD patients with more extreme patterns of brain aging, which would be important to identify as accelerated brain aging may be reversed with targeted treatment. For example, one study showed that brain-PAD was temporarily reduced by 1.1 years in healthy controls owing to the acute anti-inflammatory effects of ibuprofen^93^. Inflammatory biomarkers are commonly dysregulated in MDD and negative relationships between levels of inflammatory cytokine (e.g., interleukin-6) and cortical thickness have been found in medication-free, first-episode MDD patients^94^, suggesting that inflammation may be a common biological mechanism between MDD and brain aging. Notably, brain-PAD has been shown to be a general predictor of psychiatric and neurological disorders, with low clinical disease specificity^95^.

#### White matter microstructure

We also examined white matter microstructure in 1305 MDD patients, and 1602 healthy controls from 20 samples participating in ENIGMA MDD worldwide, again using a meta-analytic approach^38^. The ENIGMA protocol for diffusion tensor imaging (DTI)^33^ calculates fractional anisotropy (FA) for 25 atlas-defined white matter tracts of interest. FA is a commonly used measure in DTI analysis, and higher values indicate directionally constrained diffusion of water molecules within the white matter, which is mostly interpreted as higher degree of myelin integrity. In addition to FA, the ENIGMA DTI protocols also yield the following diffusivity metrics: axial diffusivity (AD), which is thought to represent the number, caliber, and organization of axons, radial diffusivity (RD), which may be a measure of myelination, and mean diffusivity (MD), which is often considered a measure of membrane density^96^. As maturation of white matter tracts continues through adolescence and young adulthood, adolescent (age ≤21 years) and adult (age >21 years) patients and controls were analyzed separately. The meta-analysis showed subtle but widespread changes in FA, with lower FA in adult MDD patients observed in 16 out of 25 ROIs (Cohen’s *d* between 0.12 and 0.26) (Fig. 3b). Adult MDD patients also showed higher RD in multiple tracts (Cohen’s *d* between 0.12 and 0.18), potentially reflecting changes in the morphology of glial cells or myelination^97,98^. These alterations in FA and RD appeared to be global effects, as after correction for average FA and RD across the white matter skeleton respectively, these effects were no longer significant. Nevertheless, and in accordance with previous studies, the strongest regional changes in FA were observed in the genu and body of the corpus callosum and the corona radiata^99,100^. The corpus callosum connects brain regions in both hemispheres, including regions involved in mood regulation such as the anterior cingulate cortices and orbitofrontal cortices, whereas the corona radiata is part of the limbic-thalamo-cortical circuitry and is also implicated in mood regulation^101^.

The effect sizes for case–control differences in adults were small, but very similar to the effect sizes reported in the meta-analysis of subcortical volume and cortical morphology^35,36^. Also, in line with the previous cortical and subcortical meta-analysis findings, the widespread alterations in FA in adult patients were driven by MDD patients with recurrent episodes (*N* = 645), as there were no significant differences between first-episode patients (*N* = 169) and controls (*N* = 816). This again suggests that these alterations may reflect the cumulative effect of stress on brain morphology, rather than a vulnerability factor for MDD, although the reduced statistical power for the first-episode patients comparison may also explain this difference. In line with the cortical meta-analysis findings, but in contrast with findings on subcortical volume, we observed lower FA in patients with an adult age of onset (*N* = 399) compared with controls (*N* = 869) (Fig. 3b), but no differences when comparing patients with an adolescent age of onset (*N* = 251) compared with controls (*N* = 853). We hypothesized that MDD may interact with the normal aging process of white matter, which was in line with findings from our diagnosis-by-age interaction analysis, in which we observed that MDD was associated with an accelerated decline in overall FA with increasing age compared to controls.

We could not replicate the case–control differences in white matter microstructure in adults in a sample from the UK Biobank (*N* = 2096 patients and 3275 healthy controls), which may be related to lower severity of MDD symptoms in the UK Biobank sample or subtle differences in image processing. In addition, there were no significant differences in FA or diffusivity measures between adolescent patients and controls in the ENIGMA MDD sample after correction for multiple testing, with smaller effects in adolescents potentially related to lower disease duration and number of episodes in adolescent patients compared with adult patients. However, we cannot rule out that our sample of adolescent participants may still have been too small to detect subtle effects (*N* = 372 patients and 290 healthy controls).

#### Sex differences in depression-related structural brain alterations

Major depression is more than twice as prevalent and the disease burden of MDD is 50% higher in females than in males^1^, which may suggest different etiological pathways to developing MDD in males and females. However, across our ENIGMA MDD studies examining subcortical, cortical, and white matter integrity differences in MDD^35,37,38,80^, we found no diagnosis-by-sex interaction effects in adult MDD patients, indicating that structural brain alterations likely do not contribute to these sex differences in MDD. In addition, even though the model fits of the brain aging models improved when trained separately in males and females, the (subtle) advanced brain aging that we observed in adults with MDD was not different for male versus female patients^91^. Nonetheless, sex differences in structural brain alterations may be present during specific sensitive periods of brain development, such as adolescence or more specifically, during puberty^102^. We did indeed found higher RD only in adolescent males with MDD, but not females, compared with adolescent controls^38^. However, we did not observe sex differences in the cortical alterations in the adolescent MDD group^36^. This could perhaps be explained by the observation that sex differences in white matter volumes increase from birth, through adolescence, until males and females reach adulthood, whereas sex differences in gray matter remain relatively stable across development^103^. Future research would also benefit from a separation between gender and sex analyses.

### Childhood maltreatment

Of central relevance to understanding the role of environmental factors contributing to neurophenotypes of MDD is research focused on childhood adversity and maltreatment. Indeed, childhood maltreatment is relatively common in the general population^104,105^ and is associated with an increased risk of a multitude of psychiatric illnesses, including MDD^104,106^. In addition to epidemiological and clinical evidence linking childhood maltreatment and MDD, recent neuroimaging studies show that brain structures affected by childhood maltreatment are also implicated in the etiology and expression of MDD symptoms^107–109^.

Two studies from the ENIGMA MDD consortium examined the effects of childhood maltreatment on brain structure in depressed and non-depressed individuals using a mega-analytic approach^75,110^. In the two largest studies to date examining associations between childhood maltreatment and brain structure, Frodl et al. and Tozzi et al. examined the association between severity of childhood maltreatment—including emotional, physical and sexual abuse, or emotional and physical neglect as assessed with the childhood trauma questionnaire (CTQ)—and brain morphometry in a total of 3036 and 3872 individuals with and without MDD, respectively. Across all individuals, and correcting for MDD diagnosis, greater exposure to childhood maltreatment was associated with lower cortical thickness of the banks of the superior temporal sulcus (STS) and supramarginal gyrus (SMG), and with lower surface area across the whole brain and in the middle temporal gyrus (Fig. 3b). Childhood maltreatment severity interacted with age such that greater severity and older age were associated with lower cortical thickness in banks of the STS, SMG, rostral anterior cingulate cortex (rACC), OFC, ACC, posterior cingulate cortex (PCC), insula, precuneus, and frontal and temporal lobe regions. This regional pattern is consistent with the cortical meta-analysis study^36^, where we found that adult patients (>21 years old), and especially those with an adult age of onset of MDD, had lower cortical thickness in the bilateral OFC, ACC, PCC, insula, frontal, and temporal lobe regions. It is thus possible, then, that in adults these depression-related cortical regions are explained by the severity of childhood maltreatment.

The effects of childhood maltreatment on subcortical structures in MDD and healthy controls, however, were distinct from the effects of MDD on subcortical structures. Notably, in females only—although the same pattern showed a trend towards significance in males—greater maltreatment severity was associated with smaller caudate volumes^110^. This result stands in contrast to the first paper from the ENIGMA MDD consortium, where we found that MDD was associated with smaller hippocampal volumes, but not smaller caudate volumes, regardless of sex. As part of the dorsal striatum, the caudate is involved in motor planning, procedural learning, and reward-based reinforcement learning^111,112^. Specifically, the caudate codes representations of expectation violation and reward prediction errors that underlie approach and avoidance behaviors and reward-based learning, all of which are significantly altered in individuals exposed to childhood adversity and maltreatment^113,114^. While keeping in mind the heterogeneity of MDD and the limitations of retrospective reports of childhood maltreatment^115^, in the context of understanding neurophenotypes of MDD thus far, our results may suggest that smaller hippocampal volumes result from (stress-related) mechanisms directly associated with MDD, whereas smaller caudate volumes may result from exposure to stress during sensitive periods of development (i.e., childhood). Of note, MDD was no longer associated with smaller hippocampal volumes when corrected for childhood maltreatment^110^, although the overall sample included in the subcortical meta-analysis^35^ was larger than the sample included in this childhood maltreatment mega-analysis and there was also a difference in the composition of the cohorts evaluated. Future longitudinal studies in youth exposed to childhood maltreatment are needed to disentangle primary consequences of childhood maltreatment on brain integrity, from secondary associations caused by prolonged stress experiences and/or the development of maltreatment-associated psychiatric diseases such as MDD.

### Suicidal thoughts and behaviors

Many individuals with MDD experience suicidal thoughts, and major depressive episodes account for at least half of suicide deaths^116^. The lifetime probability of suicide attempts is 20–25% among people with major mood disorders^117^. Prior studies had identified structural brain alterations in individuals with MDD and a history of suicidal thoughts and behaviors, with most consistent evidence for structural deficits in the ventromedial and ventrolateral PFC, dorsomedial and dorsolateral PFC, ACC, insula and posterior structures including PCC, temporal regions, and the cerebellum (for recent reviews, see^118–123^). Findings of structural alterations in subcortical regions, including the amygdala, hippocampus and striatal regions have been less-consistent across studies. However, published studies have been primarily conducted using small sample sizes (typically *N* < 50 per group). Therefore, we performed an IPD-based meta-analysis of subcortical volumes, lateral ventricle volumes, and total ICV using data from *N* = 1101 people with MDD (451 of whom exhibited suicidal ideation or behavior) and *N* = 1996 healthy controls from seven research cohorts participating in the ENIGMA MDD consortium^124^. Groups were identified based on the presence of suicidal ideation, defined as thinking about suicide or taking one’s life, but without making any specific plan or acting upon those thoughts; suicidal planning, or the systematic formulation of a program of action that has the potential to lead to a suicide attempt; and a suicide attempt, defined as any self-initiated action aimed at terminating one’s life, regardless of the method or degree of its consequences. Because the number of suicide attempters (*N* = 14) was too small to allow any cross-group comparison, the MDD individuals with suicide attempts were grouped with those MDD individuals with suicide planning into a single category (suicidal behavior).

No significant association of suicidal thoughts and behavior with any of the subcortical volumes was found^124^. MDD patients reporting suicidal plans or attempts did show a 2.87% smaller ICV (Cohen’s *d* = −0.284) than controls, but no significant differences were found when compared with the MDD patients with only suicidal ideation without a plan or those without suicidal ideation and behavior. These null findings with regard to subcortical volumes could perhaps be explained if additional involvement of subcortical regions, and especially the hippocampus^35^, in suicidal thoughts and behaviors beyond their role in MDD is subtle and only apparent in studies with very large sample sizes. Alternatively, given the highly heterogeneous nature of both MDD and suicidality, subcortical structural alterations may only become apparent in specific subgroups of people with suicidal thoughts and behaviors. Finally, it may be that cortical structural alterations play a greater role than subcortical alterations in suicidal thoughts and behaviors. Efforts to identify cortical structural alterations associated with suicidal thoughts and behaviors are currently ongoing within ENIGMA MDD in a sample with a higher prevalence of suicide attempts.

### Impact of antidepressant medication

With regard to antidepressant medication use at the time of scanning, patients taking antidepressants tended to show greater structural alterations than antidepressant-free patients, both in the subcortical and cortical ENIGMA MDD meta-analysis studies^35,36^. These findings are counterintuitive as antidepressant treatment has been associated with reduced hippocampal atrophy by putatively enhancing synaptic plasticity and neurogenesis^125^. However, as the majority of the ENIGMA MDD cohorts did not collect detailed information on the history of antidepressant use, the duration of use, time since last antidepressant treatment and dose of the antidepressant, and given the cross-sectional nature of the studies, these findings cannot be interpreted as direct effects of antidepressant medication use. MDD patients taking antidepressants at the time of scanning were likely the most severe/chronic or recurrent patients in the sample, so the results are likely to be confounded by the severity or course of the disorder. Potential neuroprotective effects of antidepressant medication are more consistent with our cortical surface area findings in adolescents with MDD, showing lower cortical surface area in several regions in antidepressant-free adolescent patients compared with healthy controls but no differences between adolescent patients taking antidepressants and healthy adolescents^36^. Confounding effects of recurrent or chronic illness were also minimized in this group given their earlier stage of illness.

In contrast to associations between antidepressant use and more pronounced cortical thickness and hippocampal volume abnormalities in adults with MDD, the meta-analysis of white matter microstructure revealed no differences between adult patients who were taking antidepressants at the time of scanning and healthy individuals^38^. Differences in white matter microstructure were, however, present in adults with MDD who were antidepressant-free at the time of scanning compared with controls^38^. This finding was unexpected as the meta-analysis of white matter microstructure and the meta-analysis of cortical thickness were both performed in a partly overlapping sample of adults with MDD with a similar prevalence of recurrent episode patients (79% versus 71%). Therefore, if the greater and more widespread cortical thickness alterations were driven by a more severe course of the disorder, a similar effect would have been expected with regard to white matter microstructure. These findings raise the question of whether antidepressant medication may have differential effects on different characteristics of the brain (e.g., cortical thickness versus surface area and white matter microstructure) within the same individuals. Effects of antidepressant medication use on measures of gray matter and white matter microstructure require further investigation in a sample with more detailed and comprehensive information on antidepressant treatment (e.g., information on history, type of antidepressant, and duration of use) and simultaneous use of other medications (e.g., atypical antipsychotics). In addition, although many human and animal studies have examined the effects of short-term antidepressant medication use on brain structure, there is limited information on the effects of long-term antidepressant medication use on brain structure. In this respect, to disentangle indirectly associated phenomena from causal effects of antidepressants, longitudinal studies are needed, preferably with a focus on long-term exposure and at different stages of brain development and aging.

## Scientific and clinical relevance

Our findings were computed from many data sets combined, which has provided a more reliable estimate of effect sizes of structural brain alterations associated with MDD than have individual small sample studies. This inclusion of large-scale, diverse samples also enabled us to calculate and report how reproducible these structural brain alterations are across data sets and how well findings generalize to cohorts with different ages of onset, duration of illness, and with different geographic origins. Critically, most of our findings were based on a meta-analytic approach, which increases rigor. Moreover, we also extend retrospective meta-analyses of published studies by including data that have not been previously published owing to publication bias and by using harmonized data processing and statistical analysis protocols across all data sets.

Our work has identified subtle structural brain alterations that are associated with specific demographic and clinical characteristics of MDD. In particular, specific features of brain structure were differentially associated with MDD at different stages of life and different stages of illness. Specifically, the associations with hippocampal and amygdala volumes/shapes and cortical surface area were documented in adults with an adolescent-onset MDD and in adolescents with MDD, respectively. In contrast, cortical thickness reductions and white matter abnormalities were associated specifically with adult-onset MDD and with older age in individuals with MDD and childhood maltreatment (Fig. 3). Moreover, the subcortical and white matter alterations found in patients with recurrent episodes compared with healthy controls, where absent in first-episode MDD patients, compared with healthy controls. These findings have generated novel hypotheses regarding different features of brain structure being involved in the onset and progression of depression at different stages of brain development and provide important directions for future research. For example, reductions in cortical surface may represent an early developing subtype of depressive disorder, potentially preceding the onset of MDD. If confirmed in future longitudinal studies, this could provide important information for development of novel prevention and early intervention strategies for depression.

Many of the structural brain alterations identified in the ENIGMA MDD studies have smaller effect sizes than had been assumed based on previously published studies, even in more homogeneous subgroups of MDD patients. However, many of the larger effect sizes observed in prior studies may have been owing to small sample sizes and publication bias. Effect sizes of neuroimaging measures have been shown to have noticeable instability up to as many as 1000–2000 subjects (e.g., see Figure S1 in Miller et al.^126^). Indeed, large-scale studies, including studies that pool existing data such as ENIGMA as well as large population-representative samples^126,127^, are beginning to show that variability in structural and functional brain imaging accounts for only a small percentage of the explained variance of clinical phenotypes. Thus, similar to genetics literature, it appears that individual measures of structural brain alterations account for limited variance in complex phenotypes such as depression.

These findings have important implications for our theoretical understanding of MDD; small effect sizes make it unlikely that MDD can be explained by a generic disease process, which is perhaps not surprising given the multi-causal nature of this highly complex disorder. In addition, from a clinical perspective, these small effect sizes may make it unlikely for individual structural brain measures to provide diagnostic biomarkers. Effect sizes between a Cohen’s *d* of 1.5 and 3 are likely to be required for a biomarker to be clinically useful, depending on the nature of the application^128^. Nonetheless, given that data available in ENIGMA MDD are cross-sectional, it remains to be elucidated whether any of these structural brain measures could serve as predictive or prognostic biomarkers, or as indices of treatment response that are related to long-term mental and physical health outcomes. Furthermore, multiple factors with small effect sizes can be combined to create a large effect. Therefore, the findings to date motivate future ENIGMA MDD studies to investigate whether the combination of different neuroimaging modalities as well as combining neuroimaging with clinical, psychosocial, and other biological data modalities (e.g., using machine-learning methods) could explain more variance in the depressive phenotype, with the ultimate goal of developing clinically useful diagnostic or predictive tools.

## Future directions

The ENIGMA MDD consortium is a dynamically evolving consortium, in which new research groups continue to join and new projects are continually being initiated. Our first studies have mainly focused on case–control differences in structural brain measures that can reliably be identified and replicated across many samples worldwide. An important next step within ENIGMA MDD is investigating higher dimensional structural brain measures (e.g., using vertex-wise or voxel-wise analysis), which may be better able to detect subtle regional structural brain alterations in MDD, with potentially larger effect sizes. Future work to identify potential histological, genetic, and environmental mechanisms underlying these structural brain alterations is also underway. Furthermore, as can be seen in Fig. 1, Asian research institutions are under-represented in ENIGMA MDD. Many research institutions in China have shared neuroimaging data from individuals with depression with the REST-meta-MDD consortium, which has recently published the first large-scale mega-analysis on resting state functional MRI data of 1300 depressed patients and 1128 healthy controls from 25 research groups in China^129^. Future collaborations between the ENIGMA MDD and REST-meta-MDD consortia will be important for identifying potential cultural differences in brain alterations associated with MDD.

Future plans of the ENIGMA MDD consortium also include (but are not limited to): (1) parsing the heterogeneity of MDD, (2) moving beyond structural brain measures to include functional brain alterations, and (3) elucidating whether the identified neuroimaging markers are unique to MDD or shared across mental disorders, which are further discussed below.

### Addressing the heterogeneity of MDD

By pooling data across many samples worldwide, the ENIGMA MDD consortium performs studies encompassing a range of depressive phenotypes—from very mild to severe and a broad range of previous treatments received. This broad spectrum of depressive phenotypes combined with a very large sample size provides the opportunity to study the phenotypic and neurobiological heterogeneity of MDD. Analyses within subtypes, rather than across a heterogeneous diagnosis-based sample, could reveal more pronounced changes in brain structure and function. For example, large samples allow the stratification or clustering patients into different subgroups while preserving sufficient statistical power within each subgroup.

In addition, heterogeneity could be addressed by examining individual differences, for which large samples are required to capture the full range of variation in the phenotype. Such approaches may reveal clues for the development of treatments tailored to subtypes or individual differences. At present, there are various ongoing ENIGMA MDD projects that aim to address this heterogeneity by examining associations between brain alterations and depressive symptom subtypes (e.g., atypical depression) and the presence or absence of phenotypes closely related to MDD (e.g., obesity).

In addition, it is also important to investigate associations with individual symptoms, as individual symptoms differ in their impact on impairment of functioning, their response to specific life events, their risk factors^130^, as well as their response to treatment (e.g., Chekroud et al^131^.). Therefore, several ongoing projects in ENIGMA MDD have taken a dimensional approach to identify neural correlates of between-subject differences in the severity of individual symptoms (e.g., insomnia, suicidality).

Moreover, the pathophysiology of MDD is also likely highly heterogeneous. Different pathophysiological mechanisms can result in similar symptoms for different individuals (equifinality) and the same underlying biological risk factors may result in a different expression of a certain disorder depending on an interaction with the environment and genetic vulnerability (multifinality). In line with recent studies using brain imaging markers to identify subtypes of MDD defined by different profiles of biological markers, so-called “biotypes” (e.g. Drysdale et al.^132^, but also see Dinga et al.^133^ for limitations associated with this approach), we aim to investigate potential biotypes and their replicability across multiple cohorts in ENIGMA MDD.

### Functional neuroimaging

An important next frontier in ENIGMA MDD will be to characterize brain functional deficits in MDD. Although the past two decades have witnessed a surge in studies on resting state fMRI, the vast heterogeneity in analysis methods, choice of seeds, templates, and parcellation atlases has yielded a patchwork landscape of results in the literature. So far, only a few resting state meta- or mega-analyses exist for major depression^129,134^, either focusing on one specific network or combining results from different analysis strategies across studies. We therefore plan to conduct a large resting state analysis by pooling data from sites across the world, assessing a range of resting state features using harmonized processing and a standard set of seeds, templates, and atlases.

In addition to rsfMRI, future endeavors will also include task fMRI. In the spirit of the Research Domain Criteria framework^135^, we will hone future analyses to task paradigms that tap into functional domains relevant to depressive symptomatology, in particular, spanning the negative valence and cognitive domains. So far, meta-analyses in MDD mainly have been based on coordinates of peak statistical difference reported in single published studies^14^, using tools such as activation likelihood estimation (ALE)^136^. As potential case–control differences with small effect sizes (typically not reported if not significant in single studies) may be omitted in such meta-analyses and previous negative findings may have not been published, it will be important to expand this work through applying IPD-based meta- and mega-analyses. Therefore, we will conduct voxel- (or vertex-)wise meta-analyses, which have been shown to be superior to coordinate-based meta-analyses^137,138^. A caveat is whether different versions of task paradigms—or even different tasks probing the same functional domain—can be combined meaningfully in a meta- or mega-analysis. This needs to be further confirmed empirically, although preliminary results from the ENIGMA task-based fMRI workgroup are promising^139^.

Variance introduced by the different scanner types and acquisition parameters at each of the contributing sites cannot be avoided, but we can overcome the apparent heterogeneity in preprocessing, feature extraction, and statistical testing of fMRI data by harmonizing software packages, preprocessing settings, task contrasts, seed masks, parcellation atlases, network templates, and statistical models across participating sites. Similarly, the same rigorous quality assessment (QA) procedures should be employed across participating sites, judging data quality against centrally defined criteria. Following the example of using standardized analytical tools and QA procedures for structural analyses in ENIGMA, such procedures and tools are currently developed within the ENIGMA task-based and ENIGMA resting state fMRI methods working groups.

### Identifying shared and unique brain alterations across mental disorders

**Fig. 4:**
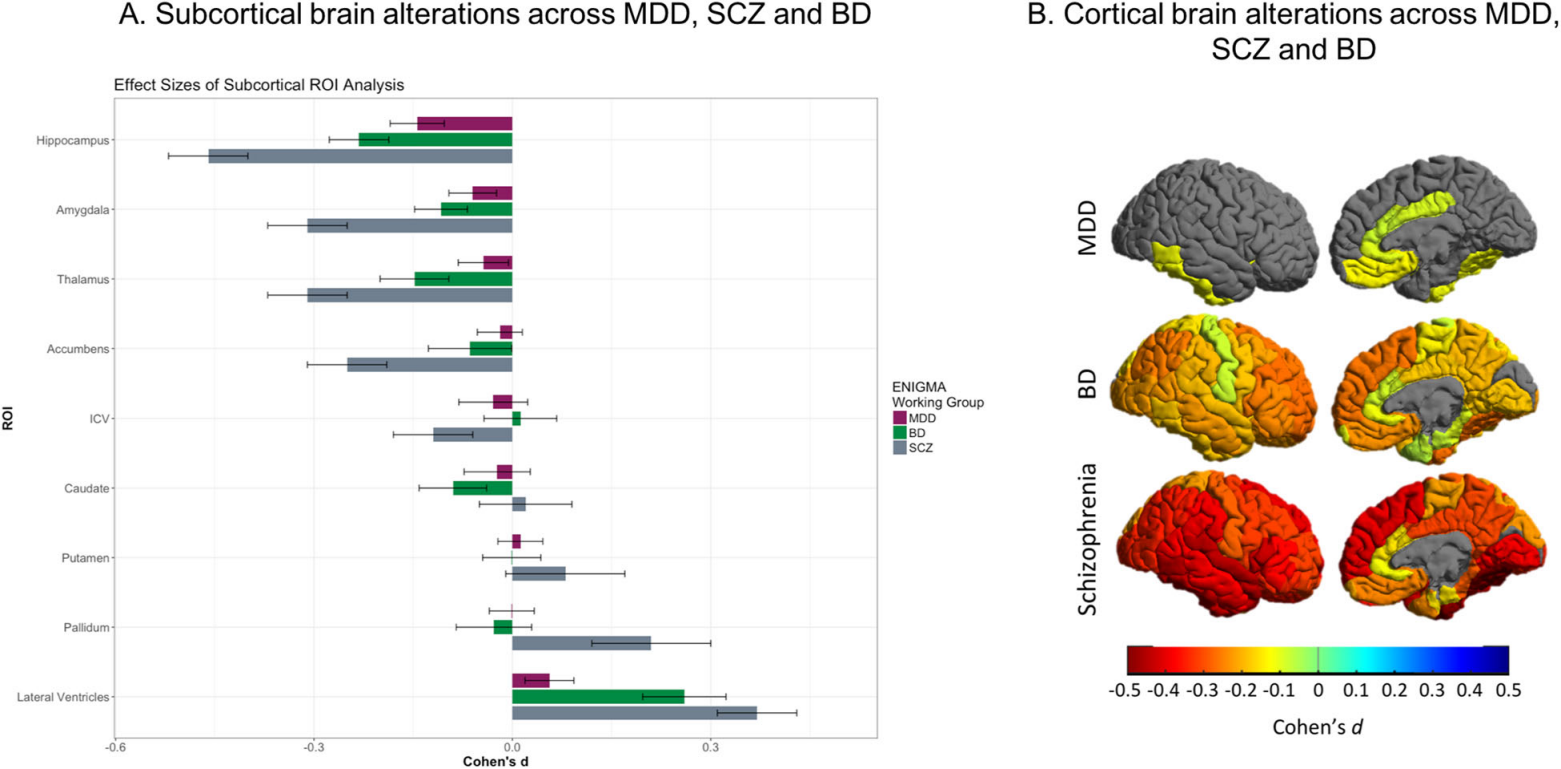
Subcortical volume and cortical thickness alterations in schizophrenia, bipolar disorder, and MDD. Results from the ENIGMA major depressive disorder (MDD), schizophrenia (SCZ), and bipolar disorder (BD) working groups suggest that there is considerable overlap in subcortical volume **a** and cortical thickness **b** alterations across these diagnostic groups. Most widespread effects and highest effect sizes were observed in SCZ (up to Cohen’s *d* 0.5), followed by BD (Cohen’s *d* 0.3), and with more local effects and lower effect sizes in MDD (Cohen’s *d* 0.15). Importantly, results displayed are based on case–control comparisons within each disorder separately and are not derived from direct comparisons between patient groups. Data were analyzed with the same harmonized methods across the disorders.

Until recently, all ENIGMA disease working groups have focused on comparisons of a single disorder with healthy individuals using neuroimaging data. Results from primary projects in ENIGMA have indicated that schizophrenia (SCZ)^80^, bipolar disorder (BD)^140^, and MDD^36^ patients are all characterized by lower prefrontal and temporal cortical thickness relative to healthy control subjects. However, effect sizes differed between disorders, the largest (up to Cohen’s *d* 0.5) having been observed in SCZ, followed by BD (Cohen’s *d* 0.3) and MDD (Cohen’s *d* 0.15). A similar gradient was observed for hippocampal volume across these disorders^35,141,142^, suggesting an “affective-psychotic severity continuum” (Fig. 4). Moreover, in ENIGMA, the obsessive–compulsive disorder (OCD) consortium likewise found lower hippocampal volume in OCD patients versus healthy controls, but this effect was at least partly driven by patients with comorbid MDD^143^, again suggestive of a shared mechanism. Notably, hippocampal volume loss was also observed in post-traumatic stress disorder even after accounting for childhood trauma^144^. Finally, the findings of structural brain alterations in people with substance use disorders from the ENIGMA Addiction consortium also overlap with our findings in MDD, showing similar effect sizes for the hippocampus, insula, and medial OFC^145^.

Given that all ENIGMA psychiatric disease working groups use the same standardized preprocessing pipelines and analysis protocols, ENIGMA is well positioned to perform cross-disorder comparison studies. Several cross-disorder initiatives are ongoing, including comparisons of brain morphology across SCZ, BD, and MDD as well as across neurodevelopmental disorders (autism spectrum disorder, attention deficit hyperactivity disorder, OCD, and Tourette’s syndrome)^146^. The presence of suicidal thoughts and behaviors and childhood maltreatment are also relevant, as these constitute transdiagnostic constructs.

## Challenges of large-scale data-sharing initiatives

Worldwide data-sharing initiatives such as ENIGMA are not without their challenges. Some of these challenges encompass ethical and computational issues with regard to data sharing, as well as science and data sharing policies that vary from one research institute to another, from country to country or even from continent to continent and may change over time. This may restrict some researchers from sharing raw neuroimaging data, although sharing de-identified, individual-level data may still be feasible. In addition, several challenges need to be addressed in translating the small to moderate effect sizes observed throughout ENIGMA MDD to the individualized and generalizable prediction of MDD-related phenotypes. The first is the need for rigorous testing and validation of predictive models in multiple independent samples. A challenge in this respect is the relative unavailability of deeply characterized phenotypes and longitudinal data. To date, ENIGMA MDD has largely relied on existing data, which implies a degree of heterogeneity with respect to phenotyping including clinical assessments, limiting the analysis of sources of clinical heterogeneity. In addition, the current focus of ENIGMA MDD is on cross-sectional studies. Consequently, our findings require further investigation in longitudinal studies to elucidate, for example, influences of brain development and aging, medication effects, and the clinical relevance of the observed structural brain alterations in MDD. Combining longitudinal samples is not without its challenges, but has already been successfully done for healthy individuals by the ENIGMA Plasticity working group^147^.

Another limitation is that neuroimaging data were collected using different MRI scanners, different sequences, different brain coverage and, for functional analyses, different paradigm versions or acquisition lengths, which may all introduce noise and further complicate the search for robust biological markers of MDD. Efforts to develop post-processing methods to reduce noise associated with differences in scanning and other characteristics between cohorts will be important, especially in the context of machine-learning analysis.

Finally, it could be the case that certain findings regarding the neurobiology of MDD will not be obtained by ever larger meta-analyses of existing samples; we may need alternative methods of data collection or new data types that are sensitive to effects that are undetected today. We stress the need for a many-pronged approach using novel data collection and the coordinated analysis of the data already available, as well as the development of new approaches.

## Conclusion

Over the past 7 years since its initiation, ENIGMA MDD has brought together research groups across the world with broad expertize to work together to gain a better understanding of brain abnormalities associated with MDD. By addressing issues of underpowered studies, our work has provided more reliable estimates of the extent of structural brain abnormalities in depression, showing that variability in structural brain alterations may only account for a small percentage of the depression phenotype. Future work is underway that aims to address the heterogeneity of depression and to integrate across data modalities to better understand the multi-causal nature of depression, with the ultimate goal to help develop or select more effective treatments for MDD.
